# Actor-critic learning-based energy optimization for UAV access and backhaul networks

**DOI:** 10.1186/s13638-021-01960-0

**Published:** 2021-04-07

**Authors:** Yaxiong Yuan, Lei Lei, Thang X. Vu, Symeon Chatzinotas, Sumei Sun, Björn Ottersten

**Affiliations:** 1 Interdisciplinary Center for Security, Reliability and Trust, University of Luxembourg, 1855 Kirchberg, Luxembourg, Luxembourg; 2 Institute for Infocomm Research, Agency for Science, Technology, and Research, Singapore , 138632 Singapore

**Keywords:** UAV, Deep reinforcement learning, User scheduling, Backhaul power allocation, Energy optimization, Actor-critic

## Abstract

In unmanned aerial vehicle (UAV)-assisted networks, UAV acts as an aerial base station which acquires the requested data via backhaul link and then serves ground users (GUs) through an access network. In this paper, we investigate an energy minimization problem with a limited power supply for both backhaul and access links. The difficulties for solving such a non-convex and combinatorial problem lie at the high computational complexity/time. In solution development, we consider the approaches from both actor-critic deep reinforcement learning (AC-DRL) and optimization perspectives. First, two offline non-learning algorithms, i.e., an optimal and a heuristic algorithms, based on piecewise linear approximation and relaxation are developed as benchmarks. Second, toward real-time decision-making, we improve the conventional AC-DRL and propose two learning schemes: AC-based user group scheduling and backhaul power allocation (ACGP), and joint AC-based user group scheduling and optimization-based backhaul power allocation (ACGOP). Numerical results show that the computation time of both ACGP and ACGOP is reduced tenfold to hundredfold compared to the offline approaches, and ACGOP is better than ACGP in energy savings. The results also verify the superiority of proposed learning solutions in terms of guaranteeing the feasibility and minimizing the system energy compared to the conventional AC-DRL.

## Introduction

Unmanned aerial vehicle (UAV)-assisted communication has been widely applied to various domains, e.g., aerial inspection, precision agriculture, traffic control, and after-disaster rescue [2]. Compared to terrestrial cellular systems, UAV-assisted systems (1) provide on-the-fly communication, which expands the coverage of ground wireless devices, and (2) have higher probability to experience Line-of-Sight (LoS) transmission, which improves channel quality. In addition, the advances of UAVs’ manufacturing technologies reduce the deployment cost of UAV networks and popularize their commercial and civilian usages [3].

However, one of the most critical issues of UAV-assisted networks is the limited on-board energy, which may shorten the UAVs’ endurance and lead to service failure. Therefore, minimizing the UAV’s energy consumption is of great importance. In [4], the authors proposed a joint power allocation and trajectory design algorithm to maximize UAV’s propulsion energy efficiency. With the consideration of both communication energy and propulsion energy of UAV, the authors in [5] and [6] proposed energy-efficient communication schemes via user scheduling and sub-channel allocation, respectively. We note that the works in [4–6] focused on the access link in UAV-assisted networks, where the UAV serves as an aerial base station (BS) that carries all the ground users’ (GUs’) requested data. In practice, due to limited storage capacity, the GU’s requested data may be not available in the UAV’s cache. When the BS in the GU’s service area is overloaded or damaged, the UAV serves as an intermediate node to acquire requested data from a remote auxiliary base station (ABS) through a backhaul link and deliver data to the GUs via access links [7]. Compared to the direct terrestrial communication between the GU and the ABS, UAV undergoes better channel conditions but with limited energy supply. Thus, it is necessary to consider energy-saving problems for backhaul-access UAV networks. In [8], an energy efficiency maximization problem was investigated via power allocation and trajectory design, where the UAV performs as a relay between ABS and GUs. The authors in [9] proposed a joint trajectory design and spectrum allocation algorithm to minimize UAV’s propulsion energy while satisfying the backhaul constraint, meaning that the transmitted data of the access link must be less than that of the backhaul link.

The user scheduling schemes in [8, 9] are based on time division multiple access (TDMA) or frequency division multiple access (FDMA) with a single-antenna UAV. However, spatial division multiple access (SDMA) mode with multiple-antenna techniques and precoding design is able to improve network capacity, thereby reducing the tasks’ completion time and total energy consumption. In [10], a non-orthogonal multiple access-based user scheduling and power allocation algorithm was proposed to minimize UAV’s transmission energy with the backhaul constraint. In [11], the authors designed a game theory-based precoding scheme for multi-antenna UAV-assisted cluster networks. To maximize the UAV’s propulsion energy efficiency, the authors in [12] proposed a power allocation scheme for multi-antenna UAV-enabled relay systems. However, the energy consumption of the backhaul link is studied to a limited extent in the above works [10–12], which is a large proportion of the total energy consumption and could be optimized by backhaul power control [13]. This motivates us to investigate an energy minimization problem, including both backhaul and access energy, in multiple-antenna UAV-assisted networks.

Optimization-based solutions, e.g., successive convex approximation [5] or Lagrangian dual method [6], might not be able to make time-efficient decisions. First, the SDMA-based transmission mode enables the UAV to serve more than one GU simultaneously, resulting in exponential growth of decision variables as well as the complexity [1]. Moreover, diversified energy models in UAV systems may lead to non-convexity in problem formulation, which makes the problem difficult to be solved optimally.

Deep reinforcement learning (DRL) learns the optimal policy from the interaction between environment and actions, instead of directly solving the optimization problem. DRL combines artificial neural networks with a reinforcement learning architecture to improve learning efficiency and solution quality. Different from deep neural networks (DNNs), DRL is not necessary to prepare a large amount of data in advance for offline training. To maximize the energy efficiency, the authors in [14] and [15] applied deep Q network (DQN) to make decisions for resource block allocation and flight path planning, respectively. DQN needs to establish a Q-table containing all the possible actions before executing the algorithm so that it is usually for the decision tasks with discrete action space and a small number of decision variables [16].

Actor-critic-based DRL (AC-DRL) can tackle both discrete and continuous action space. For the problem with continuous variables, e.g., power control, AC-DRL adopts a stochastic policy to select an action by probability. In [17], an energy-efficient UAV’s direction control policy was proposed based on AC-DRL. To minimize UAV’s energy consumption, in [18], the authors applied an AC-based deep deterministic policy gradient algorithm for UAV’s velocity and direction control. In [17, 18], multiple decision variables in the problem modelings may lead to huge action space and slow convergence (more than 1000 learning episodes). It is noted that the solution proposed in [17, 18] can be applied to only unconstrained problems. However, for general UAV-assisted networks, the optimization problems have constraints [4–9, 11–13]. Therefore, directly applying AC-DRL may not lead to a high-quality and feasible solution.

In this paper, we propose two tailored AC-DRL-based schemes: AC-based user group scheduling and backhaul power allocation (ACGP), and joint AC-based user group scheduling and optimization-based backhaul power allocation (ACGOP). The main contributions are summarized as follows:

- We formulate a non-convex mixed-integer programming (NCMIP) problem to minimize both backhaul energy and access energy in UAV-assisted networks.
- To approach the optimum, we first transform the non-linear terms to linear by piecewise linear approximation and McCormic envelopes, leading to a mixed-integer linear programming (MILP) problem, which can be solved optimally by branch and bound (B&B).
- We provide a near-optimal algorithm with lower computation time than the optimal method. First, the original NCMIP problem is relaxed to a continuous optimization problem. Second, the relaxed problem is converted to a linear programming (LP) problem by piecewise linear approximation. Then, the heuristic solutions can be obtained after taking a rounding-up operation.
- Being aware of the high-complexity optimization methods, we propose ACGP and ACGOP learning schemes. To enable the learning algorithms to adapt to the considered NCMIP, in ACGP and ACGOP, we improve the conventional AC-DRL by a set of approaches, i.e., action filtering and reward re-design, to improve learning performance and avoid infeasible solutions.
- From the numerical results, we conclude that, compared with non-learning algorithms, ACGP and ACGOP have superiority in computational time efficiency, while compared with conventional AC-DRL, ACGP and ACGOP achieve better performance in delivering feasible solutions. Experiments also show that the combined learning-optimization scheme, i.e., ACGOP, achieves better energy-saving performance than ACGP.

The rest of the paper is organized as follows. Section 2 provides the system model. In Sect. 3, we formulate the considered optimization problem and solve it by proposing an optimal algorithm and a heuristic algorithm. In Sect. 4, we resolve the problem by DRL and develop an AC-DRL-based algorithm. Numerical results are presented and analyzed in Sect. 5. Finally, we draw the conclusions in Sect. 6.

*Notations:* Some mathematical operators are defined as follows. For a vector ${\varvec{a}}$, $\Vert {\varvec{a}}\Vert$ and ${\varvec{a}}^{\text {H}}$ represent its Euclidean norm and conjugate transpose, respectively. For a matrix ${\varvec{A}}$, ${\varvec{A}}^{\text {H}}$ refers to its conjugate transpose, and ${\varvec{A}}^{\dagger }$ denotes its generalized inverse matrix. For scalars *x* and *y*, $\lceil x\rceil$ and $\lfloor x\rfloor$ means rounding-up and rounding-down operations, respectively. $\left[ x\right] ^{+}$ is equivalent to $\max \{0,x\}$. ${{\mathcal {N}}}(x,y)$ means a Gaussian distribution with a mean *x* and a variance *y*. For a random variable *X*, ${\mathbb {E}}[X]$ is the statistical expectation of *X*.

## System model

**Fig. 1:**
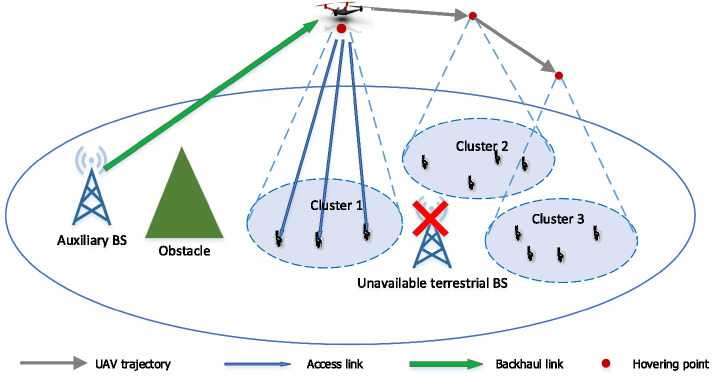
A UAV network with $N=3$ clusters

**Fig. 2:**
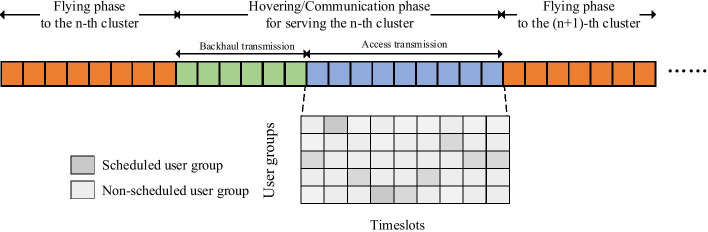
An illustration of the timeline of UAV actions

We consider a UAV-assisted communication system including both backhaul and access links, as shown in Fig. 1. In the backhaul part, a multi-antenna UAV requests data from a multi-antenna ABS which is connected to the core network. In the access network, the UAV acts as an aerial BS to serve single-antenna GUs in remote areas when the terrestrial BS in the current service area is not available, e.g., destroyed in a disaster. As the UAV operates at high altitudes, it can overcome the influence of obstacles on the ground, e.g., buildings or mountains, and has more probability to experience LoS transmission. The difference between the backhaul and access networks in channel modeling is that the former forms a MIMO system while the latter is modeled as a multi-user MISO system. When the UAV receives GUs’ data requests, it first downloads these data from a remote ABS through a backhaul link and then distributes data to GUs through access links. The GUs in the service area are divided into several clusters due to the limited communication coverage of the UAV. As an input to the UAV optimization problem, GUs clusters can be determined by two methods. One is by clustering algorithms, e.g., K-means, based on the similarity of the GUs’ distances or channel conditions. The second is simply based on the GUs association and coverage area of the damaged base stations. In this paper, the latter method is adopted. In a cluster, there exist *K* single-antenna GUs and each has $q_k$ (bits) demands. The user set is denoted as ${{\mathcal {K}}}=\{1,...,k,...,K\}$. The total demands is denoted by $D=\sum _{k=1}^{K}q_k$. In each transmission task, all the GUs’ demands need to be served within the time limitation $T_{max}$ (seconds), including the time used for acquiring data from ABS and delivering data to GUs^1^. As shown in Fig. 2, the system spectrum is reused in a TDMA fashion so that the time domain of a transmission task is divided into a sequence of timeslots ${{\mathcal {I}}}=\{1,...,i,...,I\}$, where *I* is the maximum number of timeslots, given by $\lfloor \frac{T_{max}}{\Phi }\rfloor$, and $\Phi$ (seconds) refers to the duration of each timeslot. In the access network, a timeslot accommodates multiple GUs with the SDMA transmission mode to further improve network capacity.

### Backhaul transmission

The ABS and UAV are equipped with $L_t$ and $L_r$ antennas, respectively, so that the backhaul link can be modeled as a MIMO channel. We assume that signals propagate through LoS transmission from ABS to UAV. Let ${\mathbf {G}}\in {\mathbb {C}}^{L_t\times L_r}$ be the channel matrix of the wireless backhaul link, which is determined by the spherical wave model [19] which is given by:

1 $$\begin{aligned} {\mathbf {G}} = \left[ \begin{array}{ccc} o^{-\beta }_{1,1}e^{\text {j} 2\pi f_c o_{1,1}} &{}\cdots &{}o^{-\beta }_{1,L_r}e^{\text {j} 2\pi f_c o_{1,L_r}}\\ \vdots &{}\ddots &{}\vdots \\ o^{-\beta }_{L_t,1}e^{\text {j} 2\pi f_c o_{L_t,1}} &{}\cdots &{}o^{-\beta }_{L_t,L_r}e^{\text {j} 2\pi f_c o_{L_t,L_r}} \end{array} \right] , \end{aligned}$$

where $o_{l_t,l_r}$ corresponds to the path length between the $l_t$-th transmitting antenna and the $l_r$-th receiving antenna, $f_c$ refers to the carrier frequency, and $\beta$ is the path loss exponent. The received signal at the UAV from the ABS can be described by:

2 $$\begin{aligned} {\mathbf {y}} = {\mathbf {G}}{\mathbf {x}} + {\mathbf {n}}, \end{aligned}$$

where ${\mathbf {x}}$ and ${\mathbf {n}}$ denote the transmitted signal and white Gaussian noise of the UAV, respectively. In order to maximize the backhaul capacity, we employ the water-filling-based power allocation [20]. The matrix ${\mathbf {G}}$ has a singular value decomposition (SVD):

3 $$\begin{aligned} {\mathbf {G}}={\mathbf {U}}\mathbf {\Lambda }{\mathbf {V}}^{\dagger }, \end{aligned}$$

where ${\mathbf {U}}\in {\mathbb {C}}^{L_t\times L_t}$ and ${\mathbf {V}}\in {\mathbb {C}}^{L_r\times L_r}$ are unitary matrices, and $\mathbf {\Lambda }\in {\mathbb {C}}^{L_t\times L_r}$ is a diagonal matrix whose elements are non-negative real numbers. The diagonal elements $\lambda _1,...,\lambda _L$ in $\mathbf {\Lambda }$ are the ordered singular values (from large to small) for ${\mathbf {G}}$. Under the assumption that ${\mathbf {G}}$ is a full-rank matrix, let $L=\min \{L_t,L_r\}$. We process the UAV’s received signal by:

4 $$\begin{aligned} \tilde{\mathbf {y}}={\mathbf {U}}^{\dagger }{\mathbf {y}}=\sqrt{{\mathbf {P}}}\mathbf {\Lambda }{\mathbf {V}}^{\dagger }{\mathbf {x}}+{\mathbf {U}}^{\dagger }{\mathbf {n}}, \end{aligned}$$

where $\sqrt{{\mathbf {P}}}=diag(\sqrt{p_1},...,\sqrt{p_L})$ referring to a diagonal matrix, and $p_l$ means the power allocation among the antennas. Thus, the capacity of the MIMO channel can be calculated by:

5 $$\begin{aligned} r^{bh} = B^{bh}\sum _{l=1}^L \log _2\left( 1+\frac{p_l\lambda _l^2}{\sigma ^2}\right) , \end{aligned}$$

where $B^{bh}$ is the bandwidth of the backhaul link, and $\sigma ^{2}$ is the receiver noise power of the UAV. Based on the water-filling power allocation, $p_l^* = \left[ \mu -\frac{\sigma ^2}{\lambda _l^2}\right] ^{+}$, where $\mu$ is the water-filling level [20]. Thus, the total transmit power on the backhaul is:

6 $$\begin{aligned} p^{bh}(\mu ) = \sum _{l=1}^{L}p_l^* = \sum _{l=1}^{L}\left[ \mu -\frac{\sigma ^2}{\lambda _l^2}\right] ^{+}. \end{aligned}$$

The achievable rate of the backhaul can be rewritten as:

7 $$\begin{aligned} r^{bh}(\mu ) = B^{bh}\sum _{l=1}^L\left[ \log _2\left( \frac{\mu \lambda _l^2}{\sigma ^2}\right) \right] ^{+}. \end{aligned}$$

At a timeslot, the backhaul transmission energy and the achievable transmitted data volume are:

8 $$\begin{aligned} e^{bh}(\mu )&= \Phi p^{bh}(\mu ), \end{aligned}$$

9 $$\begin{aligned} d^{bh}(\mu )&= \Phi r^{bh}(\mu ). \end{aligned}$$

### Access transmission

From Fig. 2, in the access transmission, the shaded block indicates that the user is scheduled. We define the scheduled users as a user group. Therefore, the maximum number of candidate groups can be calculated by $G = \sum _{l=1}^{L_r}\frac{K!}{l!(K-l)!}$, which increases exponentially with *K*. The group combination is ${{\mathcal {G}}}=\{1,...,g,...,G\}$. Toward eliminating multi-user interference within a group, minimum mean square error (MMSE) precoding is applied [21]. The signal is propagated between the UAV and GUs via a LoS channel. We denote $K_g$ and ${{\mathcal {K}}}_g$ as the number and set of users in group *g*, and ${\mathbf {h}}_{k,g} \in {\mathbb {C}}^{L_r\times 1}$ as the channel vector for user $k \in {{\mathcal {K}}}_g$, which is expressed as:

10 $$\begin{aligned} {\mathbf {h}}_{k,g} = \left[ \iota ^{-\beta }_{k,g,1}e^{\text {j} 2\pi f_c \iota _{k,g,1}}, \cdots , \iota ^{-\beta }_{k,g,L_r}e^{\text {j} 2\pi f_c \iota _{k,g,L_r}}\right] , \end{aligned}$$

where $\iota _{k,g,l_r}$ means the distance between the UAV’s $l_r$-th antenna and the *k*-th GU of the *g*-th group. We form ${\mathbf {H}}_{g} = \left[ {\mathbf {h}}_{1,g},...,{\mathbf {h}}_{K_g,g}\right]$ as the channel matrix of group *g*. Based on the MMSE, the precoding vector ${\mathbf {w}}_{k,g} \in {\mathbb {C}}^{L_r\times 1}$ can be calculated by:

11 $$\begin{aligned} {\mathbf {w}}_{k,g} = \frac{\tilde{{\mathbf {h}}}_{k,g}}{\Vert \tilde{{\mathbf {h}}}_{k,g}\Vert }, \end{aligned}$$

where $\tilde{{\mathbf {h}}}_{k,g}$ is to the *k*-th column of the MMSE precoding matrix ${\mathbf {H}}_{g}^{\text {H}}(\sigma _{k,g}^2{\mathbf {I}}+{\mathbf {H}}_{g}{\mathbf {H}}_{g}^{\text {H}})^{-1}$, $\sigma _{k,g}^2$ is the noise power for user $k \in {{\mathcal {K}}}_g$ and ${\mathbf {I}}$ is an identity matrix. Since the UAV’s transmit power is a constant selected from 0.1 W to 10 W in practical UAV application [22], we assume the transmit power for user *k* in group *g* is fixed, denoted as $p_{k,g}$. The received signal at GU $k \in {{\mathcal {K}}}_g$ is given by:

12 $$\begin{aligned} y_{k,g}&=\sqrt{p_{k,g}}{\mathbf {h}}_{k,g}^{\text {H}}{\mathbf {w}}_{k,g} x_{k,g} + \sum _{j\in {{\mathcal {K}}}_g\setminus \{k\}} \sqrt{p_{j,g}}{\mathbf {h}}_{k,g}^{\text {H}}{\mathbf {w}}_{j,g} x_{j,g}\nonumber \\&\quad +n_{k,g},\,\,k \in {{\mathcal {K}}}_g,\,g \in {{\mathcal {G}}}. \end{aligned}$$

where $x_{k,g}$ and $n_{k,g}$ denote the transmitted signal and white Gaussian noise of GU $k \in {{\mathcal {K}}}_g$. According to (12), we obtain the SINR of GUs $k \in {{\mathcal {K}}}_g$ as:

13 $$\begin{aligned} {S\!I\!N\!R}_{k,g} = \frac{p_{k,g} |{\mathbf {h}}_{k,g}^{\text {H}}{\mathbf {w}}_{k,g}|^2}{\sum _{j\in {{\mathcal {K}}}_g\setminus \{k\}} p_{j,g} |{\mathbf {h}}_{k,g}^{\text {H}}{\mathbf {w}}_{j,g}|^2+\sigma _{k,g}^2}, \end{aligned}$$

Thus, the transmitted data volume for GU $k\in {{\mathcal {K}}}_g$ and the transmission energy for group *g* can be expressed as:

14 $$\begin{aligned} d_{k,g}&= \Phi r_{k,g} = \Phi B^{ac} \log _2\left( 1+{S\!I\!N\!R}_{k,g}\right) , \end{aligned}$$

15 $$\begin{aligned} e_{g}&= \Phi p_g = \Phi \sum _{k\in {{\mathcal {K}}}_g} p_{k,g} , \end{aligned}$$

where $B^{ac}$ is the bandwidth of the access link.

### UAV energy model

The propulsion power can be modeled as a function with regards to the flying velocity *U* [23], which is given by:

16 $$\begin{aligned} {{\mathcal {P}}}(U) =&P_0\left( 1+\frac{3U^2}{U_{tip}^2}\right) +P_1\left( \sqrt{1+\frac{U^4}{4U_{ind}^4}}-\frac{U^2}{2U_{ind}^2}\right) ^{\frac{1}{2}}\nonumber \\&\quad +\frac{1}{2}\varrho _{1}\varrho _{2}U^3, \end{aligned}$$

where $P_0$ and $P_1$ are the blade profile power and induced power in hovering status, respectively. $U_{tip}$ and $U_{ind}$ refer to the tip speed of the rotor blade and mean rotor induced velocity, respectively. $\varrho _1$ is the parameter related to the fuselage drag ratio, rotor solidity, and the rotor disc area. $\varrho _2$ is denoted as the air density.

In the hovering phase, the UAV flies circularly around a hovering point with a small radius. To minimize the hovering power, the hovering velocity is given by:

17 $$\begin{aligned} U^{hov}={{\,\mathrm{argmin}\,}}_{U\ge 0} {{\mathcal {P}}}(U). \end{aligned}$$

Therefore, the hovering energy is only related to the hovering time. In the flying phase, the energy consumption with flying distance *S* is expressed as $\frac{S{{\mathcal {P}}}(U)}{U}$. When the flying path is predetermined, *S* is a constant parameter such that the flying velocity that minimizes the flying energy is:

18 $$\begin{aligned} U^{fly}={{\,\mathrm{argmin}\,}}_{U\ge 0} \frac{{{\mathcal {P}}}(U)}{U}. \end{aligned}$$

Both $U^{hov}$ and $U^{fly}$ can be obtained by graph-based numerical methods [24]. Therefore, the hovering power $p^{hov}$ and flying power $p^{fly}$ are ${{\mathcal {P}}}(U^{hov})$ and ${{\mathcal {P}}}(U^{fly})$. Because the UAV suspends data transmission when flying between the clusters in the fly-hover-communicate protocol [5], the minimum flying energy is $\frac{S{{\mathcal {P}}}(U^{fly})}{U^{fly}}$.

### UAV flying path selection and fly-hover-communicate protocol

In the considered scenario, the UAV visits and serves each cluster’s data requests in a sequential manner according to the predetermined trajectory and visiting orders. Before taking off, the UAV pre-optimizes the trajectory according to different requirements at the dock station. We keep the trajectory design flexible. For example, if the UAV task is time-critical, the flying path can be determined by the clusters’ priorities, e.g., the higher-priority cluster is served first. If the task is energy-critical, we apply Dijkstra’s algorithm to obtain the shortest or minimal cost path which is mainly adopted in this paper [25].

The timeline of UAV actions is depicted in Fig. 2. According to the fly-hover-communicate protocol, the UAV stops transmitting data when flying [5]. The UAV first experiences the flying phase before arriving at the hovering center of the target cluster. Then, the UAV hovers at the cluster and delivers data to the GUs, which enables equivalent hovering time and communication time. When the transmission task in the current cluster is completed, the UAV flies to the next cluster.

The main notations are summarized in Table 1.

**Table 1:** Summary of symbols and notations

Notation	Description
$L_t, L_r$	Number of transmitting, receiving antennas in UAV
$K_n, {{\mathcal {K}}}_n$	Number and set of users in cluster *n*
$G_n, {{\mathcal {G}}}_n$	Number and set of groups in cluster *n*
$K_{g,n}, {{\mathcal {K}}}_{g,n}$	Number and set of users in group *g* of cluster *n*
$q_{k}, D$	Demands of user *k* and total demands
$T_{max}$	Time limitation for each task (in seconds)
$I, {{\mathcal {I}}}$	Number and set of timeslots in each frame
$\Phi$	Duration of each timeslot (in seconds)
$\mu$	Water-filling level
$e^{bh}(\mu )$	Backhaul energy
$d^{bh}(\mu )$	Transmitted data in backhaul link
$e^{hov}$	Hovering energy at each timeslot
$e_g$	Communication energy for group *g* at each timeslot
$d_{k,g}$	Transmitted data for user *k* in group *g* at each timeslot

## Problem formulation and Heuristic approach

### Problem formulation

Our goal is to minimize the total system energy consumption via a joint design for user-timeslot scheduling and backhaul power allocation subject to the users’ quality of service requirements. The total energy consumption consists of four parts: (1) the flying energy, (2) the hovering energy, (3) the backhaul transmission energy, and (4) the access transmission energy. As analyzed in the previous section, the flying energy is independent from the scheduling and power transmission decisions and hence can be skipped in the joint design. On the other hand, the hovering energy is determined by the transmission time and hence needs to be optimized.

We denote a set of binary variables indicating timeslot allocation as follows:

$$\begin{aligned} \alpha ^{ac}_{g,i}=&\left\{ \begin{array}{ll} 1, &{} \text {group } g \in {{\mathcal {G}}}\text {is scheduled at timeslot} i, \\ 0, &{} \text {otherwise}. \end{array} \right. \\ \alpha ^{bh}_{i}=&\left\{ \begin{array}{ll} 1, &{} \text {backhaul link is scheduled at timeslot} i, \\ 0, &{} \text {otherwise}. \end{array} \right. \end{aligned}$$

Then joint design of timeslot allocation (via $\alpha ^{ac}_{g,i}, \alpha ^{bh}_i$) and backhaul power optimization (via $\mu$) for energy minimization can be formulated as follows:

19a $$\begin{aligned}&{\mathcal {P}}_1:\ \ \ \ \ \nonumber \\&\min \limits _{\alpha ^{ac}_{g,i}, \alpha ^{bh}_{i}, \mu } \,\, \sum _{i=1}^{I}\alpha ^{bh}_{i}\left( e^{bh}(\mu )+e^{hov}\right) +\sum _{i=1}^{I}\sum _{g=1}^{G}\alpha ^{ac}_{g,i}\left( e_{g}+e^{hov}\right) \end{aligned}$$

19b $$\begin{aligned} s.t.\quad&\sum _{i=1}^{I}\sum _{g=1}^{G}\alpha ^{ac}_{g,i}d_{k,g}\ge q_k,\,\forall k \in {{\mathcal {K}}}, \end{aligned}$$

19c $$\begin{aligned}&d^{bh}(\mu )\sum _{i=1}^{I}\alpha ^{bh}_{i} \ge D, \end{aligned}$$

19d $$\begin{aligned}&\alpha ^{bh}_{i}+\sum _{g=1}^{G}\alpha ^{ac}_{g,i}\le 1, {\,\forall i\in {{\mathcal {I}}}}, \end{aligned}$$

19e $$\begin{aligned}&\mu \le u_{max}, \end{aligned}$$

19f $$\begin{aligned}&\alpha ^{ac}_{g,i}, \in \{0,1\} ,\,\forall g\in {{\mathcal {G}}},\,i\in {{\mathcal {I}}}, \end{aligned}$$

19g $$\begin{aligned}&\alpha ^{bh}_{i} \in \{0,1\} ,\,\forall i\in {{\mathcal {I}}}, \end{aligned}$$

where $e^{bh}(\mu )$ and $e_g$ are given in (8) and (15), respectively, and $e^{hov}=\Phi \cdot p^{hov}$ is the hovering energy at each timeslot.

In (19a), the first summation represents the transmission and hovering energy spent on the backhaul, and the second summation is the energy consumed on the access links. Note that we optimize water-filling level $\mu$ instead of directly optimizing backhaul power $p^{bh}$ since $p^{bh}$ depends on $\mu$ based on Eq. (6). Constraints (19b) guarantee that each GU’s request is satisfied in the access network. Constraint (19c) states that contents delivered through the backhaul should accommodate the total demands from the GUs. Constraint (19d) is to avoid concurrent transmission of the backhaul and access links. Constraint (19e) upper bounds the water-filling level to $u_{max}$, which is the maximal water-filling level under the backhaul’s limited transmit power. Constraints (19f) and (19g) confine variables $\alpha ^{ac}_{g,i}$ and $\alpha ^{bh}_{i}$ to binary.

Due to the non-convex items $e^{bh}(\mu )\alpha ^{bh}_{i}$ and $d^{bh}(\mu )\alpha ^{bh}_{i}$, ${\mathcal {P}}_1$ is a NCMIP problem which is difficult to obtain the optimal solution. One method to solve this problem is to apply a piecewise linear approximation to linearize non-linear functions, i.e., $e^{bh}(\mu )$ and $d^{bh}(\mu )$ [28]. Thus, the approximations of $e^{bh}(\mu )\alpha ^{bh}_{i}$ and $d^{bh}(\mu )\alpha ^{bh}_{i}$ have a form of bilinear function, which can be transformed to linear problems by using the McCormick envelopes [26]. The resulting problem is an integer linear programming (ILP) problem, which can be solved optimally by the B&B method [27]. When the number of linear pieces is sufficient in fitted functions and the bounds of the McCormick envelopes are sufficiently tight, the solutions can approach the global optimum. However, the operations of relaxation and approximation bring about high computation time (minutes level) which is unaffordable in practice.

### Heuristic approach

To reduce the computation time of the problem ${\mathcal {P}}_1$, we propose a heuristic algorithm. First, we consider an extreme condition $\Phi \rightarrow 0$, such that ${\mathcal {P}}_1$ can be relaxed to a continuous optimization problem ${\mathcal {P}}_2$ in (22a). After relaxation, the allocated time for group *g* and the backhaul link are continuous values:

20 $$\begin{aligned} \tau _g&= \lim _{\Phi \rightarrow 0} \Phi \sum _{i=1}^{T_{max}/\Phi } \alpha ^{ac}_{g,i}, \end{aligned}$$

21 $$\begin{aligned} \tau ^{bh}(\mu )&= D/r^{bh}(\mu ). \end{aligned}$$

${\mathcal {P}}_2$ can be formulated as follows:

22a $$\begin{aligned} \min \limits _{\tau _g,\,\mu } \quad&{{\mathcal {F}}}(\mu )+\sum _{g=1}^{G}\tau _g\left( p_g+p^{hov}\right) \end{aligned}$$

22b $$\begin{aligned} s.t.\,&\sum _{g=1}^{G}\tau _g d_{k,g} = q_k,\,\forall k \in {{\mathcal {K}}}, \end{aligned}$$

22c $$\begin{aligned}&\tau ^{bh}(\mu ) + \sum _{g=1}^{G}\tau _g \le T_{max}, \end{aligned}$$

22d $$\begin{aligned}&\tau _g > 0,\,\forall g \in {{\mathcal {G}}}, \end{aligned}$$

22e $$\begin{aligned}&\sigma ^2/\lambda _1^2<\mu \le \mu _{max}, \end{aligned}$$

where

23 $$\begin{aligned} {{\mathcal {F}}}(\mu )=&\tau ^{bh}(\mu )\cdot \left( p^{bh}(\mu )+p^{hov}\right) \nonumber \\ \overset{{\begin{array}{c} \text {Eq.}(6)\\ \text {Eq.}(7) \end{array}}}{=}&\frac{D}{B^{bh}}\cdot \frac{\sum _{l=1}^{L}\left( \mu -\frac{\sigma ^2}{\lambda _l^2}\right) ^{+}+p^{hov}}{\sum _{l=1}^L\left[ \log _2\left( \frac{\mu \lambda _l^2}{\sigma ^2}\right) \right] ^{+}}. \end{aligned}$$

By fitting ${{\mathcal {F}}}(\mu )$ and $\tau ^{bh}(\mu )$ with piecewise linear approximations, ${\mathcal {P}}_2$ can be approximated as a linear programming (LP) problem, which can be solved by classical algorithms such as simplex method [28]. In practice, when $\Phi >0$, ${\mathcal {P}}_2$ provides a lower bound of ${\mathcal {P}}_1$ and variables $\tau _1,...,\tau _g$ are integer multiples of $\Phi$. Thus, we take a rounding-up operation for post-processing, which introduces errors but makes the solutions of ${\mathcal {P}}_2$ feasible. We summarize the proposed heuristic algorithm in Alg. 1.

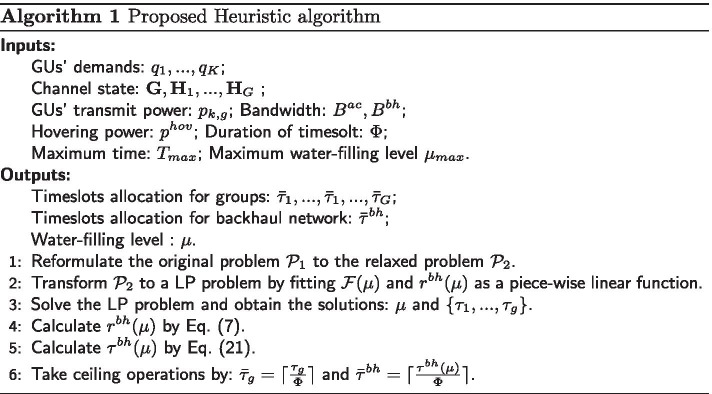



When $\Phi$ is sufficiently small, i.e., the solution of ${\mathcal {P}}_2$ approaches the optimal solution of ${\mathcal {P}}_1$ and the proposed heuristic method provides near-optimal solutions. The heuristic algorithm is more efficient than the optimal algorithm as solving the relaxed continuous problem is easier than solving its original integer programming problem. However, ${\mathcal {P}}_2$ is still suffered from high computation complexity as the number of variables is $G+1$, which exponentially increases with the number of GUs. This limits its application practice when the number of users is large or the latency requirement is stringent.

## AC overview and the proposed solutions

Being aware of the high computation complexity of the iterative optimal and suboptimal algorithms, We develop ACGP and ACGOP toward real-time applications.

### AC-DRL framework

To make the paper self-contained, we provide a brief overview of the adopted AC-DRL framework first. Basic RL is modeled as a Markov decision process (MDP) with three elements: state, action and reward. At each time step *t*, the current environment is represented as a state $s_t$. The agent takes an action $a_t$ based on $s_t$ and a policy. Then a reward $r_t$ is received by the agent and the next state $s_{t+1}$ can be observed. By collecting the tuple $\{s_t, a_t, r_t, s_{t+1}\}$, the agent updates the policy iteratively with value-based or policy-based methods. The goal of an RL agent is to learn a policy that maximizes the expected cumulative reward. In DRL, the policy or other learned functions are approximated as a neural network to deal with the high-dimensional state space and improve the learning efficiency. AC is one of the DRL frameworks, which integrates the strengths of both value-based and policy-based methods [16]. AC-DRL split the learning agent into two components, where the actor is responsible for updating policies and making decisions while the critic is used for evaluating the decisions by value functions.

For the actor, the stochastic policy is applied, which is denoted as $\pi (a|s_t)$ representing the probability of taking action *a* under state $s_t$. Usually, we model $\pi (a|s_t)$ as Gaussian distribution with a mean $\psi (s_t)$ and a variance $\chi (s_t)$ [29]. At each learning step *t*, an action $a_t$ is taken by following the policy $\pi (a|s_t)$. After that, the agent receives a reward $r_t$ as the feedback. The objective of AC-DRL is to maximize the cumulative reward so that the loss function of the actor can be defined as:

24 $$\begin{aligned} J = {\mathbb {E}}[-Q^{\pi }(s_t,a_t)], \end{aligned}$$

where $Q^{\pi }(s_t,a_t)={\mathbb {E}}_{\pi }[\sum _{t'=t}^\infty \gamma ^{t'-t}r_t'|s_t,a_t]$, representing a Q-value function with a discount factor $\gamma$. The critic is to evaluate the quality of the action by estimating the current Q-value. Temporal difference (TD) learning can be applied for Q-value estimation with high learning efficiency [16]. In TD learning, the TD error is the difference between the TD target $r_t+Q^{\pi }(s_{t+1},a_{t+1})$ and the estimated Q-value $Q^{\pi }(s_t,a_t)$. The loss function of the critic is the square of TD error:

25 $$\begin{aligned} L = {\mathbb {E}}\left[ (r_t+\gamma Q^{\pi }(s_{t+1},a_{t+1}))-Q^{\pi }(s_t,a_t)\right] ^2. \end{aligned}$$

To update the policy and Q-value, we use parameterized functions, i.e., $\psi _{\varvec{\theta }_t}(s_t)$, $\chi _{\varvec{\theta }_t}(s_t)$ and $Q_{\varvec{\omega }_t}(s_t,a_t)$, to approximate $\pi (a|s_t)$ and $Q^{\pi }(s_t,a_t)$:

26 $$\begin{aligned}&\pi (a|s_t)\approx \, \pi _{\varvec{\theta }_t}(a|s_t)\sim {{\mathcal {N}}}(\psi _{\varvec{\theta }_t}(s_t), \chi _{\varvec{\theta }_t}(s_t)), \end{aligned}$$

27 $$\begin{aligned}&Q^{\pi }(s_t,a_t)\approx \, Q_{\varvec{\omega }_t}(s_t,a_t), \end{aligned}$$

where $\varvec{\theta }_t$ and $\varvec{\omega }_t$ are the parameters of the approximators. Based on the fundamental results of the policy gradient theorem [16], the gradient of $J(\varvec{\theta }_t)$ and $L(\varvec{\omega }_t)$ are given by:

28 $$\begin{aligned} \nabla _{\varvec{\theta }}J(\varvec{\theta }_t)&= {\mathbb {E}}\left[ -\nabla _{\varvec{\theta }}\log \pi _{\varvec{\theta }_t}(a_t|s_t)Q_{\varvec{\omega }_t}(s_t,a_t)\right] , \end{aligned}$$

29 $$\begin{aligned} \nabla _{\varvec{\omega }}L(\varvec{\omega }_t)&= {\mathbb {E}} \left[ 2L(\varvec{\omega }_t)\nabla _{\varvec{\omega }}\left( Q_{\varvec{\omega }_t}(s_{t+1},a_{t+1})-Q_{\varvec{\omega }_t}(s_t,a_t)\right) \right] . \end{aligned}$$

The update rules for $\varvec{\theta }_t$ and $\varvec{\omega }_t$ can be derived based on gradient descend:

30 $$\begin{aligned} \varvec{\theta }_{t+1} =&\varvec{\theta }_{t} - \rho \nabla _{\varvec{\theta }} J(\varvec{\theta }_{t}), \end{aligned}$$

31 $$\begin{aligned} \varvec{\omega }_{t+1} =&\varvec{\omega }_t - \rho \nabla _{\varvec{\omega }} L(\varvec{\omega }_t), \end{aligned}$$

where $\rho$ refers to the learning rate.

However, approximating $Q^\pi (s_t,a_t)$ directly brings about a large variance on gradient $\nabla _{\varvec{\theta }}J(\varvec{\theta }_t)$, resulting in poor convergence [30]. To reduce the variance, we estimate a V-value function $V^\pi ({\varvec{s}}_t)={\mathbb {E}}_{\pi }\left[ \sum _{t'=t}^\infty \gamma ^{t'-t}r_t'|s_t\right]$ instead of Q-value. Based on TD learning and parameterized V-value $V_{\varvec{\omega }_t}(s_t)$, the loss function of the critic can be expressed as:

32 $$\begin{aligned} L(\varvec{\omega }_t) = {\mathbb {E}}[\delta _{_V}(\varvec{\omega }_t)]^2 = {\mathbb {E}}[r_t+\gamma V_{\varvec{\omega }_t}(s_{t+1})-V_{\varvec{\omega }_t}(s_t)]^2. \end{aligned}$$

In addition, the TD error $\delta _{_V}(\varvec{\omega }_t)$ provides an unbiased estimation of Q-value [30]. Thus, we can rewrite Eq. (28) and Eq. (29) by:

33 $$\begin{aligned} \nabla _{\varvec{\theta }} J(\varvec{\theta }_t) =&{\mathbb {E}}\left[ \nabla _{\varvec{\theta }}\log (\pi (a_t|s_t;\varvec{\theta }_t))Q^{\pi }(s_t,a_t)\right] \nonumber \\ =&{\mathbb {E}}\left[ \nabla _{\varvec{\theta }}\log (\pi (a_t|s_t;\varvec{\theta }_t))\delta _{_V}(\varvec{\omega }_t)\right] , \end{aligned}$$

34 $$\begin{aligned} \nabla _{\varvec{\omega }}L(\varvec{\omega }_t) =&{\mathbb {E}} \left[ 2L(\varvec{\omega }_t)\cdot \nabla _{\varvec{\omega }}\left( V_{\varvec{\omega }_t}(s_{t+1})-V_{\varvec{\omega }_t}(s_t)\right) \right] \end{aligned}$$

In this paper, we apply DNNs as the approximators. The tuple $\{s_t,s_{t+1},r_t,\delta _{_V}(\varvec{\omega }_t)\}$ is stored in a repository over the learning process. At each learning step, a batch of tuples will be extracted as the training data for parameter updating.

### The proposed ACGP and ACGOP

We first reformulate P1 by defining states, actions, and rewards, such that an RL framework can apply. Next, we propose two AC-based solutions with highlighting the differences from conventional AC-DRL and tailored design for solving ${\mathcal {P}}_1$. In a learning episode, we denote the learning steps range from $t=1$ to $t=t_{e}$, where $t_{e}$ represents the last step when any termination condition reaches. We set the termination conditions by:

- The GUs’ requests have been completed.
- The service runs out of time.

Based on the AC-DRL framework, we consider two schemes: (1) A straightforward learning approach ACGP, i.e., the agent makes decisions for all the variables. (2) A combined AC learning and simple optimization approach, i.e., ACGOP.

For ACGP, the system states $s_t$ are jointly determined by the undelivered demands $b_{k,t}$ and the remaining timeslots $\eta _t$:

35 $$\begin{aligned} s_t = \{b_{1,t},...,b_{K,t}, \eta _t\}. \end{aligned}$$

The undelivered demands $b_{k,t}$ is the residual data to be transmitted to GU *k* at timeslots *t*. The actions $a_t$ in ACGP are corresponding to the decision variables in ${\mathcal {P}}_1$. When $t=1$, the agent predicts the water-filling level, i.e., $a_t=\mu$. The backhaul power $p^{bh}(a_t)$ and backhaul transmission rate $r^{bh}(a_t)$ can be calculated by Eq.(6), Eq.(7). Then, the backhaul energy is expressed as:

36 $$\begin{aligned} e^{bh}(a_t) = {\bar{\tau }}^{bh}(a_t) \left( p^{bh}(a_t)+p^{hov}\right) , \end{aligned}$$

where ${\bar{\tau }}^{bh}(a_t)=\lceil D/r^{bh}(a_t)\rceil$. When $t=2,...,t_e$, the agent makes the decisions for user scheduling in the access network. The action $a_t=g$, representing the index of the selected user group. The expressions of the state transition are given by:

37 $$\begin{aligned} b_{k,t+1} =&\left\{ \begin{array}{ll} q_{k}, &{} \,t=1,\\ b_{k,t} - d_{k,a_t}, &{} \,t=2,...,t_e. \end{array} \right. \end{aligned}$$

38 $$\begin{aligned} \eta _{t+1} =&\left\{ \begin{array}{ll} I - {\bar{\tau }}^{bh}(a_t), &{}\,t=1,\\ \eta _t-1,&{}\,t=2,...,t_e. \end{array} \right. \end{aligned}$$

The reward function $r_t$ is commonly related to the objective of the original problem. For example, $r_t=-e_t$ is widely adopted for min-energy problems [31], where $e_t$ is the energy consumed at step *t*, given by:

39 $$\begin{aligned} e_t=\left\{ \begin{array}{ll} e^{bh}(a_t), &{}\,t=1,\\ e_{a_t}+e^{hov}, &{}\,t=2,...,t_e. \end{array}\right. \end{aligned}$$

Note that, In the simulation $-e_t$ will be treated as a benchmark. A tailored reward function for ACGP and ACGOP can be found in (46).

In ACGOP, we observe that when user scheduling is fixed, the remaining backhaul power allocation becomes a single-variable optimization problem that is computationally light. Thus, the agent in ACGOP only takes actions for user scheduling while the backhaul power is determined by an efficient golden-section search approach. Specifically, the state $s_t$ keeps the same as in ACGP. When $t=1,...,t_{e}$, the learning agent makes decision for user scheduling, i.e., $a_t=g$. The expressions of state transition can be rewritten as:

40 $$\begin{aligned} b_{k,t+1} =&\left\{ \begin{array}{ll} q_{k} - d_{k,a_t}, &{} \,t=1,\\ b_{k,t} - d_{k,a_t}, &{} \,t=2,...,t_{e}. \end{array} \right. \end{aligned}$$

41 $$\begin{aligned} \eta _{t+1} =&\left\{ \begin{array}{ll} I - 1, &{}\,t=1,\\ \eta _t-1,&{}\,t=2,...,t_{e}. \end{array} \right. \end{aligned}$$

When a termination condition is reached, i.e., $t=t_e$, if $\eta _t\le 0$, then the solutions are not feasible, otherwise $\eta _t$ can be regarded as the available number of timeslots for backhaul transmission. Since the user scheduling is obtained by the learning agent, the original problem can be reduced to a single-variable power control problem ${\mathcal {P}}_3$:

42a $$\begin{aligned}&\min \limits _{\frac{\sigma ^2}{\lambda _1^2}<\mu \le \mu _{max}} {{\mathcal {F}}}(\mu ) \end{aligned}$$

42b $$\begin{aligned}&s.t. \tau ^{bh}(\mu ) = \Phi \eta _{t_e}, \end{aligned}$$

#### **Lemma 1**

*Assume* $\frac{\sigma ^2}{\lambda _1^2}<1$, ${{\mathcal {F}}}(\mu )$ *is a unique function with a unique minimum point in* $\left[ \frac{\sigma ^2}{\lambda _{1}^2}, +\infty \right]$.

#### *Proof*

See appendix 7.1. $$\square$$

**Fig. 3:**
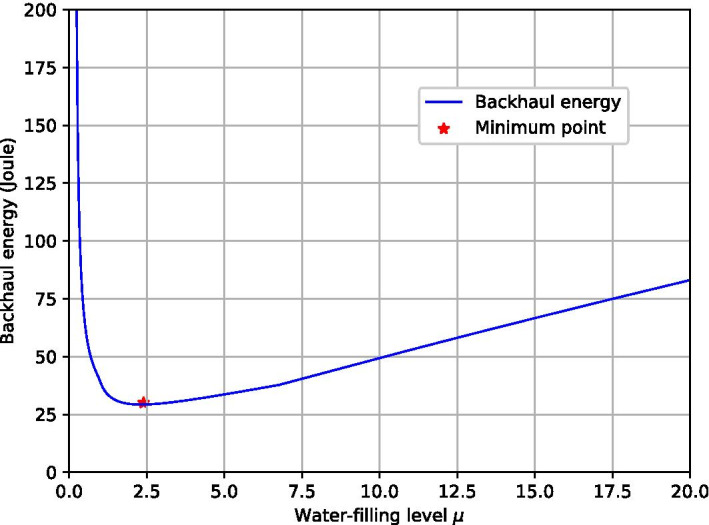
Function graph of ${{\mathcal {F}}}(\mu )$

Figure 3 illustrates the function graph of ${{\mathcal {F}}}(\mu )$. Based on Lemma 1, the optimal value $\mu ^*$ can be quickly found by golden section search [32]. After that, the backhaul energy $e^{bh}(\mu ^*)$ can be calculated by Eq. (36). The energy consumption at each time step is rewritten as:

43 $$\begin{aligned} e_t=\left\{ \begin{array}{ll} e_{a_t}+e^{hov},&{}\,t=1,...,t_e-1,\\ e_{a_t}+e^{hov}+e^{bh}(\mu ^*),&{}\,t=t_e, \end{array}\right. \end{aligned}$$

We observe that conventional AC-DRL may have limitations on dealing with ${\mathcal {P}}_1$. First, the decision variables in ${\mathcal {P}}_1$ are both continuous and discrete. Thus, we need to map the stochastic policy in AC-DRL to the corresponding action space. Second, the action spaces is huge due to the combinatorial nature of ${\mathcal {P}}_1$. Searching in such a huge space may reduce learning efficiency and solution quality. Third, conventional AC-DRL may converge to an infeasible solution without tailored reward design. In this paper, we propose a set of approaches to address the above issues.

#### Action mapping

Denote ${\hat{a}}_t$ as the original action selected by the stochastic policy $\pi (a|s_t)$. Since $\pi (a|s_t)$ follows Gaussian distribution, ${\hat{a}}_t$ is a continuous value on $[-\infty , +\infty ]$. We introduce two mapping functions:

44 $$\begin{aligned}&{{\mathcal {M}}}_1(x) = \min \{\max \{-\kappa ,0\},\kappa \}, \end{aligned}$$

45 $$\begin{aligned}&{{\mathcal {M}}}_2(x) = \lceil \frac{\kappa +{{\mathcal {M}}}_1(x)}{2\kappa /G}\rceil . \end{aligned}$$

${{\mathcal {M}}}_1(x)$ maps *x* to a continuous space $[-\kappa , \kappa ]$, where $\kappa$ is a positive parameter, while ${{\mathcal {M}}}_2(x)$ maps *x* to a discrete space ${{\mathcal {G}}}=\{1,2,...,G\}$. In order to map ${\hat{a}}_t$ to the corresponding action space, we define the after-mapped action $a_t$ as:

46 $$\begin{aligned} \bullet&\,\text {ACGP}: \,\,a_t = \left\{ \begin{array}{ll} \frac{\mu _{max}({{\mathcal {M}}}_1({\hat{a}}_t)+\kappa )}{2\kappa },&{}\,t=1,\\ {{\mathcal {M}}}_2({\hat{a}}_t),&{}\,t=2,...,t_e. \end{array}\right. \end{aligned}$$

47 $$\begin{aligned} \bullet&\,\text {ACGOP}: \,\,a_t={{\mathcal {M}}}_2({\hat{a}}_t),\,t=1,...,t_e. \end{aligned}$$

#### Action filtering

The size of discrete space ${{\mathcal {G}}}$ increases exponentially with the number of users. To confine the action space, we eliminate a considerable number of redundant actions which bring no benefit to rewards. Specifically, the redundant actions refer to scheduling the groups that contain demand-satisfied GUs. Therefore, at the beginning of each step, we take an action filtering operation to find which GUs’ demands have been satisfied and remove the corresponding groups. As a result, the action space decreases gradually over the learning steps, thereby improving the search efficiency and the solution quality.

#### Reward design

All the constraints in ${\mathcal {P}}_1$ except (19b) can be met by properly defining actions and states. The constraints (19b) cannot be guaranteed as the commonly used reward function, i.e., $r_t=-e_t$, purely minimizes energy, and the GU’s demand is not taken into account. We re-design a tailored reward function. First, if the after-learned policy is infeasible at the end of each episode, the agent will get a penalty $-\zeta$ which is negative [33]. Second, an extra reward $\epsilon \sum _{k=1}^K d_{k,a_t}$ will be added to $r_t$. That is, the reward enforces the actor to deliver more data to meet GUs’ demands. However, transmitting more data results in more energy consumption. In this case, we can decrease the weight factor $\epsilon$ to control energy growth. The re-designed reward is expressed as:

48 $$\begin{aligned} r_t=\left\{ \begin{array}{ll} -\zeta , &{}\text {if }\,t=t_e\,\text {and }\,\sum _{k=1}^{K}b_{k,t} >0 \\ -e_t+\epsilon \sum _{k=1}^K d_{k,a_t}, &{}\text {otherwise} \end{array}\right. \end{aligned}$$

In Alg. 2, we summarize the pseudo-code of ACGOP. Analogous to ACGOP, Alg. 2 can apply to ACGP by replacing Eq.(47), Eq. (43), Eq. (40) and Eq. (41) with Eq. (46), Eq. (39), Eq. (37) and Eq. (38), respectively.

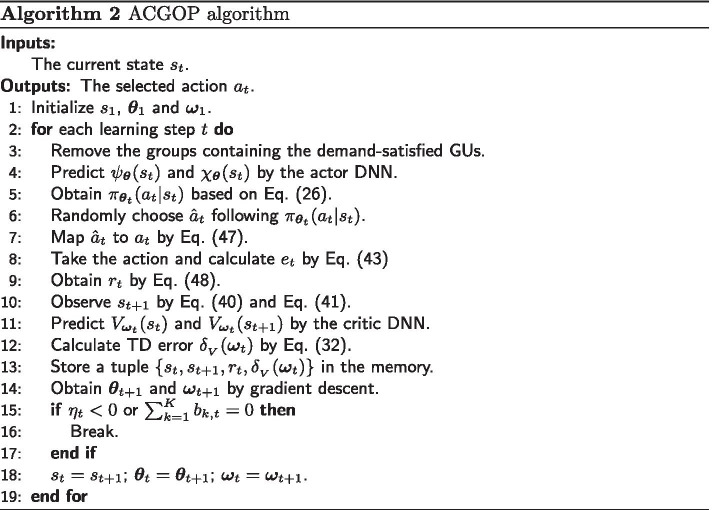



The significance of the proposed ACGOP and ACGP lies at the practical applying. The optimization tasks in a UAV-aided communication system are typically with realistic constraints and strict computational delay requirements. Compared to offline optimization approaches, ACGOP and ACGP provide online learning and timely energy-saving solutions, and achieves a good trade-off between solution quality and computational time. In addition, unlike conventional DRL methods, ACGOP combines AC learning and optimization to improve the solution quality.

## Numerical results

In this section, we evaluate the performance of the proposed solutions and other three non-learning benchmarks:

- Optimal approach (OPT): McCormick envelopes + B&B (refer to Section 3).
- Prop-HEU: Near-optimal algorithm in Alg. 1.
- Semi-orthogonal user scheduling-based heuristic algorithm (SUS-HEU) [34]: Applying SUS for user scheduling and solving ${\mathcal {P}}_3$ backhaul for power allocation.

In addition, we simulate two conventional AC-DRL schemes based on [31] for performance comparison.

### Parameter settings

The parameter setting is similar to that in [12]. We consider both the ABS and UAV are equipped with $L_t=L_r=3$ antennas. The backhaul channel matrix ${\mathbf {G}}$ and the access channel vector ${\mathbf {h}}_{k,g}$ are obtained by Eq. (1) and Eq. (10), respectively, with the carrier frequency $f_c=2.4$ (GHz) and the path loss exponent $\beta =2.6$. In the access link, the GUs are randomly scattered and separated into $N=3$ clusters. In each cluster, the number of GUs is up to $K=10$. The GUs’ demands are randomly selected from the set {3, 3.5, 4, 4.5, 5} (Gbits). We assume the bandwidth for the ABS and UAV are $B^{bh}=1$ (GHz) and $B^{ac}=0.05$ (GHz) [35]. The maximum water-filling level $\mu _{max}$ is set to 10 units. The UAV’s hovering power $p^{hov}$ and GUs’ transmit power $p_{k,g}$ is 5 (Watt) and 2 (Watt), respectively. The noise power in UAV $\sigma ^2$ and GUs $\sigma _{k,g}^2$ are -87.49 (dB) and -116.98 (dB). The duration of timeslot $\Phi$ is set as 0.1 (s).

Two fully connected DNNs are employed as the actor and the critic. The adopted parameters in ACGOP and ACGP are summarized in Table 2.

**Table 2:** Parameters in ACGOP and ACGP

Parameters	Actor	Critic
Number of hidden layers	3	3
Number of nodes/layer	300	300
Activation function (hidden layers)	ReLU	ReLU
Activation function (output layer)	Sigmoid	None
Learning rate $\rho$	0.001	0.001
Loss function	Eq. (24)	Eq. (25)
Optimizer	Adam	Adam
Batch size	64	64
Discount factor $\gamma$	0.9	
Size of repository	10,000 tuples	
Number of learning episodes	400	
Software platform	Python 3.6 with	
	TensorFlow 0.12.1	

### Results and analysis

**Fig. 4:**
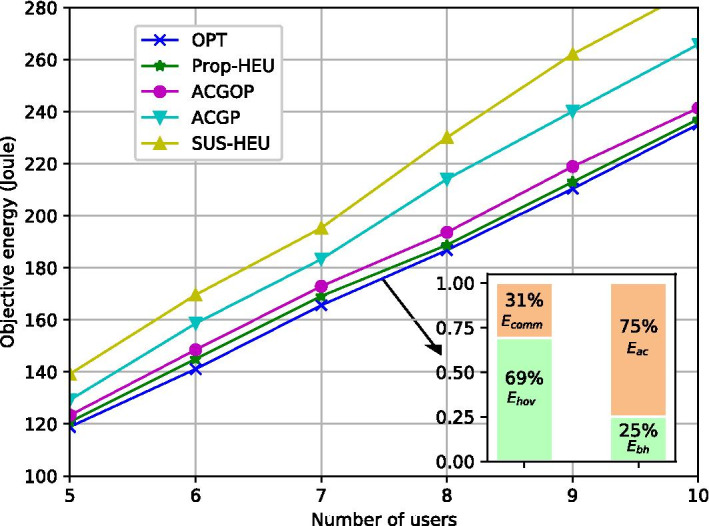
Objective energy vs. *K* ($T_{max}=16s$)

We compare the performance of the algorithms in terms of energy minimization and computation time. Figure 4 shows the objective energy with the number of users *K*. We can observe that ACGOP has 3.97% gap to the optimum, while for ACGP, the gap increases to 10.27%. Prop-HEU obtains a near-optimal solution with 1.61% average gap but requires much more computation time, e.g., see Fig. 6. SUS-HEU results in the highest energy consumption among all the schemes due to its inappropriate grouping strategy in energy savings. In addition, by averaging the results from the OPT algorithm, the sub-figure in Fig. 4 illustrates the proportion of the communication and hovering energy, and the percentage of the access and backhaul energy. The majority energy consumption is from serving access links, while the backhaul energy takes up around 25% which is a non-negligible part. The communication energy consumed on backhaul and access links accounts for 31% of the total energy while the proportion of hovering energy is 69%.

**Fig. 5:**
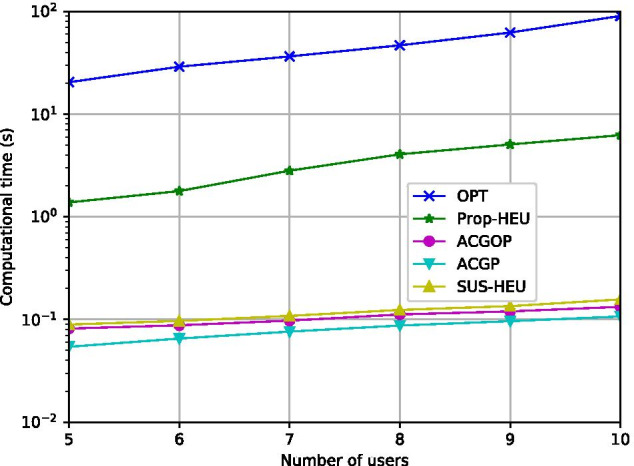
Objective energy vs. $T_{{max}}$ ($K=7$)

**Fig. 6:**
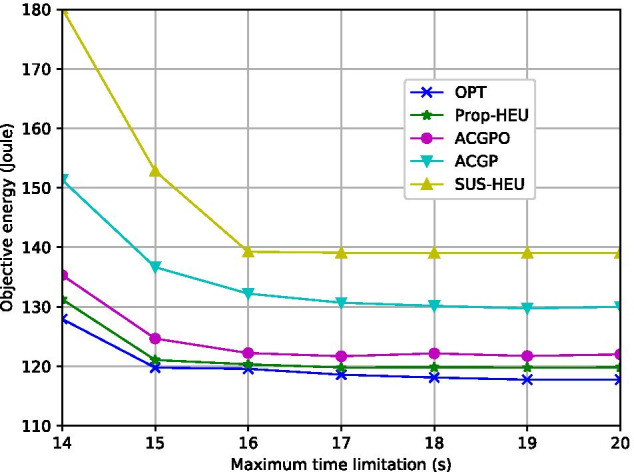
Computational time vs. *K* ($T_{max}=16s$)

Figure 5 demonstrates the total energy consumption with respect to $T_{max}$. When $T_{max}$ increases from 14 (s) to 17 (s), the energy consumption reduces by 10.43%, 12.34%, and 15.31% for Prop-HEU, ACGP, and ACGOP, respectively. This is because, in the access network, a small $T_{max}$ may enforce more GUs to share the same timeslot, which increases inter-user interference as well as the precoding energy. On the other hand, in the backhaul network, the system needs to allocate more backhaul power to satisfy the backhaul constraint within a very limited time. When the transmission time is sufficient, e.g., $T_{max}>$17 (s), the min-energy points in all the schemes are achieved.

Figure 6 compares the computation time with respect to *K*. The computation time refers to the time from giving inputs to algorithms until receiving the results. From Fig. 6, the computation time of OPT and Prop-HEU grows exponentially with *K*. When *K*=10, the computation time reaches 11 (s) and 90 (s), respectively. ACGOP, ACGP, and SUS-HEU can provide online solutions by applying the after-learned DRL policy or low-complexity SUS strategy to avoid directly solving complex optimization problems, thereby saving tenfold to hundredfold computation time compared with OPT and Prop-HEU. The average computation time of the three algorithms is relatively close. However, by recalling the energy-saving performance, ACGOP saves 8.21% and 15.28% energy compared to ACGP and SUS-HEU, respectively.

**Fig. 7:**
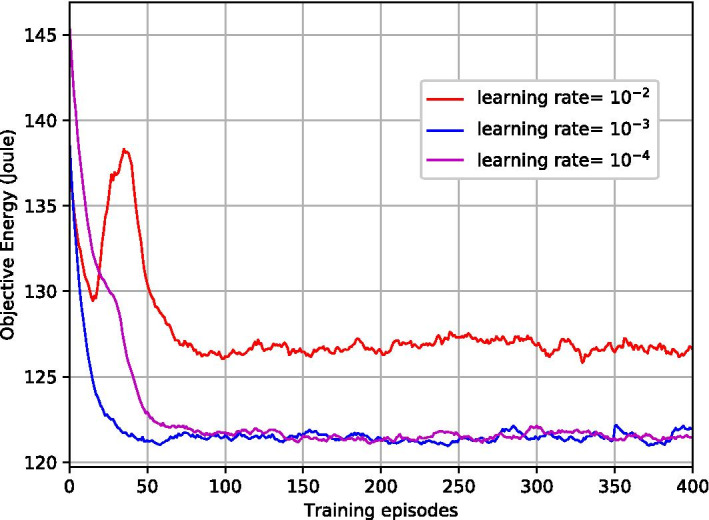
Objective energy with different learning rate

**Fig. 8:**
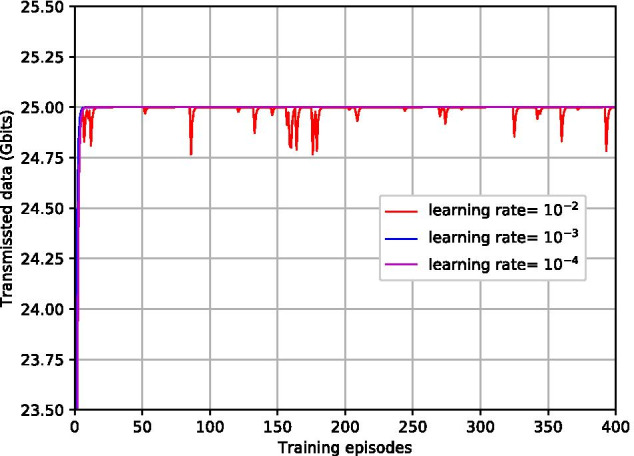
Transmitted data with different learning rate

Figures 7 and 8 illustrate the impacts of different learning rates $\rho$ for ACGOP on the performance of convergence and feasibility. From Fig. 7, we can obverse that the objective energy converges over the learning episodes. The convergence speed in the case of $\rho =10^{-3}$ is faster than that of $\rho =10^{-4}$, whereas, when $\rho$ increases to $10^{-2}$, the curve has large fluctuations and the energy at the convergence is higher than that of $\rho =10^{-3}$ and $\rho =10^{-4}$. Figure 8 depicts the total transmitted data over learning episodes. When $\rho =10^{-3}$ and $\rho =10^{-4}$, the two curves are overlapped and the after-converged solutions for both are feasible, i.e., the transmitted data are equal to the demands. But for $\rho =10^{-2}$, the feasibility cannot be guaranteed. Therefore, to achieve a fast learning speed while ensuring the feasibility of the solutions, the learning rates need to be appropriately selected. Taking ACGOP as an example, ACGP has the same tendency.

**Fig. 9:**
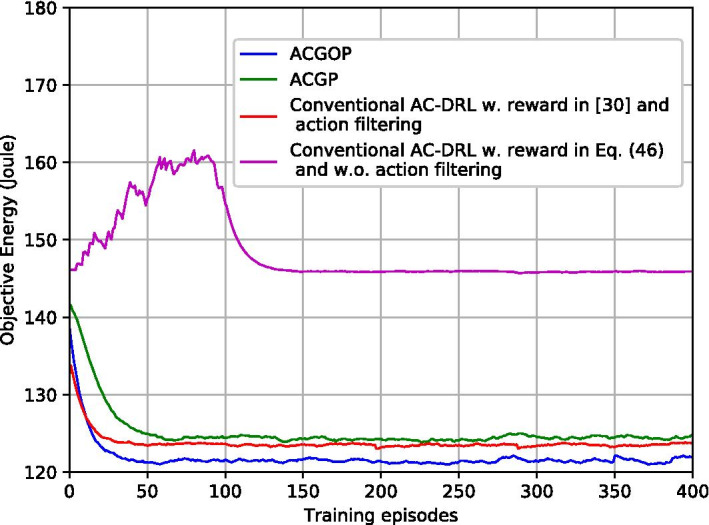
Objective energy with different AC-DRL methods

**Fig. 10:**
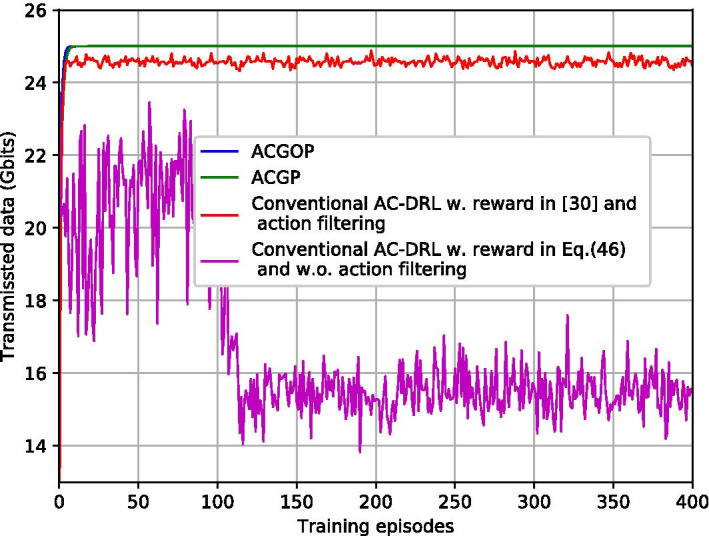
Transmitted data with different AC-DRL methods

Figures 9 and 10 compare the proposed solutions with conventional AC-DRL. From Fig. 9, ACGOP, ACGP and conventional AC-DRL with the reward in [31] and action filtering have similar performance in energy minimization. Conventional AC-DRL with the reward in Eq. (48) and without action filtering performs badly, which has slow convergence speed and high after-converged energy. Moreover, Fig. 10 demonstrates that neither the Conventional AC-DRL schemes can guarantee feasibility. The reason is that the reward in [31] is only related to the objective function but fails to consider the constraints of the problem. For AC-DRL without action filtering, a huge space may lead to low exploration efficiency and degraded performance.

**Fig. 11:**
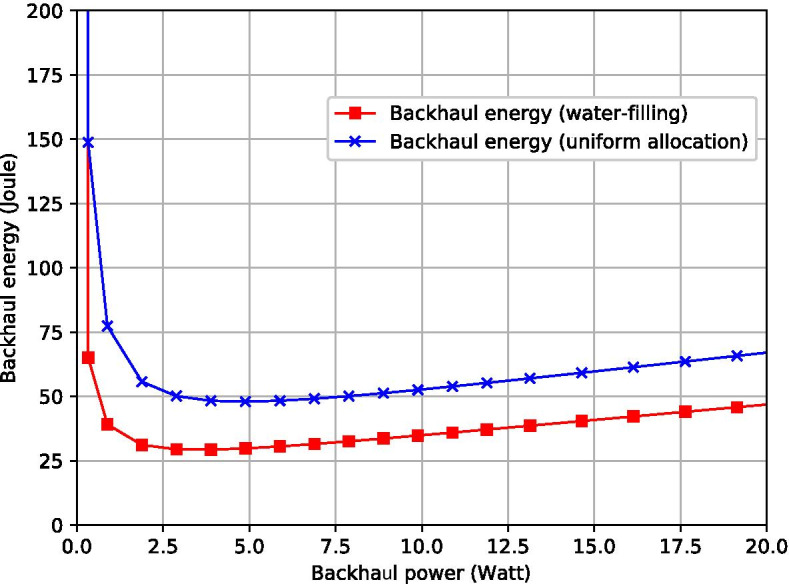
Backhaul energy vs. backhaul power

In Fig. 11, the backhaul energy, referring to the consumed communication and hovering energy due to serving backhaul, is influenced by backhaul power. In general, power optimization is needed because either lower or higher backhaul power could possibly increase energy consumption. The former could largely prolong the hovering time thus lead to a surge in energy consumption, while the latter reduces the hovering time and energy but may result in higher communication energy consumption. The minimum backhaul energy can be achieved via optimizing total backhaul power, and the water-filling-based power allocation is more energy-saving than other schemes. It can be found that the backhaul energy of the water-filling-based scheme is 40.35% lower than that of the uniform allocation scheme on average. This is because the water-filling method is able to maximize the capacity for the MIMO system. With a given total power $p^{bh}$, the water-filling-based scheme has a higher transmission rate and less transmission time $\tau ^{bh}$ than other schemes. Thus, the backhaul transmission energy $p^{bh}\tau ^{bh}$ is reduced.

## Conclusion

In this paper, we studied a joint user-timeslot scheduling and backhaul power allocation problem to minimize the energy consumption of UAV-assisted communication systems. We developed an optimal method and a heuristic algorithm as the non-learning benchmarks. Due to the high computation time, the above methods cannot provide real-time solutions. We then proposed two learning schemes, i.e., ACGP and ACGOP, based on actor-critic deep reinforcement learning. Different from conventional AC-DRL, the proposed ACGOP combines AC and optimization to accelerate learning performance. In addition, we design a set of approaches, such as action filtering and reward re-design, to reduce huge action space and guarantee feasibility. Numerical results demonstrated that ACGOP and ACGP improve computational efficiency and guarantee solution feasibility. Simulations also showed that ACGOP achieves better energy-saving performance than ACGP.

An extension of the current work is to investigate the robustness of the communication links. Considering link failure probability and allowing re-transmission, we can develop an energy-saving and robust joint user group scheduling and re-transmission scheme for UAV networks.

## Figures and Tables

**Fig. 12:**
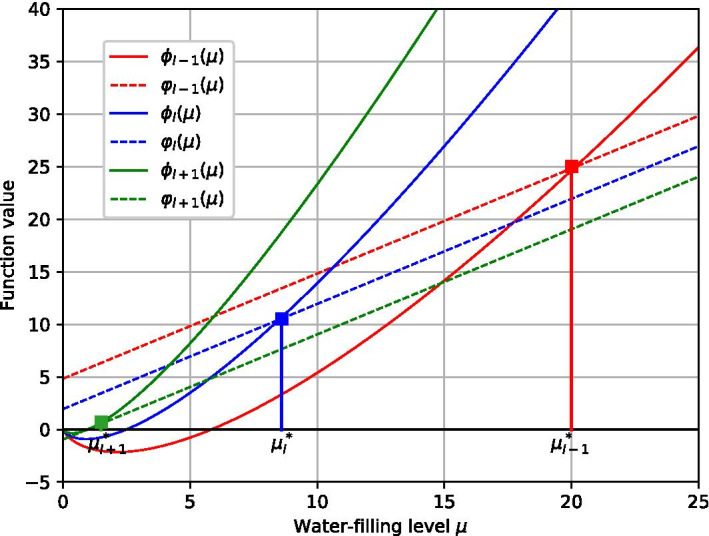
Function graphs of $\phi _l(\mu )$ and $\varphi _{l}(\mu )$

## Data Availability

The codes for generating the results are online available at the link: https://github.com/ArthuretYuan.

## Appendix

### Proof of Lemma 1

${{\mathcal {F}}}(\mu )$ can be expressed as a piecewise function:

49 $$\begin{aligned} {{\mathcal {F}}}(\mu )= f_l(\mu ),\,\,\mu \in \left[ \frac{\sigma ^2}{\lambda _l^2}, \frac{\sigma ^2}{\lambda _{l+1}^2} \right) ,\,l = 1,...,L, \end{aligned}$$

where $f_l(\mu ) = \frac{D}{B^{bh}}\cdot \frac{\mu -a_l+c_l}{\log _2(\mu )+b_l}$, $a_l=\frac{1}{l}\sum _{l'=1}^{l}\frac{\sigma ^2}{\lambda _{l'}^2}$, $b_l=\frac{1}{l}\sum _{l'=1}^{l}\log _2\left( \frac{\sigma ^2}{\lambda _{l'}^2}\right)$, $c_l = \frac{p^{hov}}{l}$ and $\lambda _{L+1}=0$. The function $f(\mu )$ can prove to be continuous but not differentiable at the breakpoints between adjacent intervals. We define $\phi _l(\mu )=\mu (\log _2(\mu )+b_l)$ and $\varphi _l(\mu )=\mu -a_l+c_l$. The first derivative and second derivative of $f_l(\mu )$ are given by:

50 $$\begin{aligned} f_l'(\mu )=\frac{D}{B^{bh}}\cdot \frac{\ln 2\cdot \phi _l(\mu )-\varphi _l(\mu )}{\ln 2\cdot \phi _l^2(\mu )/\mu }, \end{aligned}$$

Based on Eq. (50), we can derive:

51 $$(1)\;\,if\,\,\mu _{l}^{*} < \frac{{\sigma ^{2} }}{{\lambda _{l}^{2} }},\,\,f_{{l^{\prime}}} (\mu ) > 0,\mu \in \left[ {\frac{{\sigma ^{2} }}{{\lambda _{l}^{2} }},\frac{{\sigma ^{2} }}{{\lambda _{{l + 1}}^{2} }}} \right),$$

52 $$(2)\;\,if\,\,\mu _{l}^{*} \ge \frac{{\sigma ^{2} }}{{\lambda _{{l + 1}}^{2} }},\,\,f_{{l^{\prime}}} (\mu ) < 0,\mu \in \left[ {\frac{{\sigma ^{2} }}{{\lambda _{l}^{2} }},\frac{{\sigma ^{2} }}{{\lambda _{{l + 1}}^{2} }}} \right),$$

53 $$(3)\;\,if\,\,\frac{{\sigma ^{2} }}{{\lambda _{l}^{2} }} \le \mu _{l}^{*} < \frac{{\sigma ^{2} }}{{\lambda _{{l + 1}}^{2} }},\,\,f^{\prime}_{l} (\mu )\left\{ {\begin{array}{*{20}l} < 0, & \mu \in \left[ {\frac{{\sigma ^{2} }}{{\lambda _{l}^{2} }},\mu _{l}^{*} } \right), \\ = 0, & \mu = \mu _{l}^{*} , \\ > 0, & \mu \in \left( {\mu _{l}^{*} ,\frac{{\sigma ^{2} }}{{\lambda _{{l + 1}}^{2} }}} \right), \\ \end{array} } \right.$$

where $\mu _l^*$ is the point that satisfies $\ln 2\cdot \phi _l(\mu _l^*)=\varphi _l(\mu _l^*)$. Since $\lambda _l > \lambda _{l+1}$, we can derive that $a_l < a_{l+1}$, $b_l < b_{l+1}$, $c_l > c_{l+1}$ and $\mu _l^* > \mu _{l+1}^*$ by graphical method, as shown in Fig. 12.

Recalling the precondition that $$\frac{\sigma ^2}{\lambda _1^2}<1$$, it is not difficult to prove that $$\mu _1^*>\frac{\sigma ^2}{\lambda _1^2}$$. Then, $${{\mathcal {F}}}'(\frac{\sigma ^2}{\lambda _1^2})=f'_1(\frac{\sigma ^2}{\lambda _1^2})<0$$ based on Eq.(52) and Eq.(53). Moreover, $${{\mathcal {F}}}'(\mu )=f'_{L}(\mu )>0$$ when $$\mu > \max \{\mu _{L}, \frac{\sigma ^2}{\lambda _{L}^2}\}$$. Thus, there must exist minimum points in $$\left[ \frac{\sigma ^2}{\lambda _1^2}, +\infty \right)$$ on $${{\mathcal {F}}}(\mu )$$. We assume a minimum point $$\mu ^{**}$$ is located in $$\left[ \frac{\sigma ^2}{\lambda _l^2}, \frac{\sigma ^2}{\lambda _{l+1}^2} \right)$$. There are two situations:

- $$\mu ^{**} \in \left( \frac{\sigma ^2}{\lambda _l^2}, \frac{\sigma ^2}{\lambda _{l+1}^2} \right)$$. In this case, $$\mu ^{**}=\mu _l^*$$. On one hand, we can derive that $$\mu _{L}^{*}<\cdots<\mu _{l+1}^{*}<\mu _{l}^{*}<\frac{\sigma ^2}{\lambda _{l+1}^2}<\cdots <\frac{\sigma ^2}{\lambda _{L}^2}$$, i.e., $$\mu _{m}^*<\frac{\sigma ^2}{\lambda _{m}^2},\,m=l+1,...,L$$. Based on Eq. (51) and Eq.(53), it can be concluded:
  54 $$\begin{aligned} {{\mathcal {F}}}'(\mu )>0, \mu \in \left( \mu _l^*, +\infty \right) . \end{aligned}$$
  On the other hand, we can also obtain that $\mu _{1}^{*}>\cdots>\mu _{l-1}^{*}>\mu _{l}^*>\frac{\sigma ^2}{\lambda _{l+1}^2}>\frac{\sigma ^2}{\lambda _{l}^2}>\cdots >\frac{\sigma ^2}{\lambda _{2}^2}$, i.e., $\mu _{m}^*>\frac{\sigma ^2}{\lambda _{m+1}^2}, \,m=1,...,l-1$. Based on Eq. (52) and Eq. (53), it can be concluded:
  55 $$\begin{aligned} {{\mathcal {F}}}'(\mu )<0, \mu \in \left[ \frac{\sigma ^2}{\lambda _{1}^2}, \mu _l^* \right) . \end{aligned}$$
  Therefore, $$\mu ^{**}=\mu _l^*$$ is the only minimum point on $${{\mathcal {F}}}(\mu )$$.
- $$\mu ^{**} = \frac{\sigma ^2}{\lambda _l^2}$$. In this case, we can derive that $$\mu _{L}^{*}<\cdots<\mu _{l+1}^{*}<\mu _{l}^{*}<\frac{\sigma ^2}{\lambda _{l}^2}<\frac{\sigma ^2}{\lambda _{l+1}^2}<\cdots <\frac{\sigma ^2}{\lambda _{L}^2}$$, i.e., $$\mu _{m}^*<\frac{\sigma ^2}{\lambda _{m}^2},\,m=l,...,L$$, and $$\mu _{1}^{*}>\cdots>\mu _{l-1}^{*}>\mu _{l}^*>\mu _{l-1}^{*}>\frac{\sigma ^2}{\lambda _{l}^2}>\frac{\sigma ^2}{\lambda _{l-1}^2}>\cdots >\frac{\sigma ^2}{\lambda _{2}^2}$$, i.e., $$\mu _{m}^*>\frac{\sigma ^2}{\lambda _{m+1}^2}, \,m=1,...,l-1$$. Based on Eq. (51) and Eq. (52), we can conclude:
  56 $$\begin{aligned} {{\mathcal {F}}}'(\mu )&>0, \mu \in \left( \frac{\sigma ^2}{\lambda _{l}^2}, +\infty \right) , \end{aligned}$$
  57 $$\begin{aligned} {{\mathcal {F}}}'(\mu )&<0, \mu \in \left[ \frac{\sigma ^2}{\lambda _{1}^2}, \frac{\sigma ^2}{\lambda _{l}^2} \right) . \end{aligned}$$
  Therefore, $$\mu ^{**}= \frac{\sigma ^2}{\lambda _l^2}$$ is the only minimum point.

Thus, the conclusion.

## Abbreviations

UAV: Unmanned aerial vehicle
LoS: Line of Sight
GU: Ground user
TDMA: Time division multiple access
FDMA: Frequency division multiple access
SDMA: Spatial division multiple access
DRL: Deep reinforcement learning
DNN: Deep neural network
AC-DRL: Actor-critic-based DRL
NCMIP: Non-convex mixed-integer programming
MILP: Mixed-integer linear programming
ILP: Integer programming
LP: Linear programming
B&B: Branch and bound
MMSE: Minimum mean square error

## Footnotes

1 The time and energy consumed on sending requests from GUs to UAV are not considered in this paper, since they are negligible compared to those on content delivery.
